# Supplementary material for: Evolutionary dynamics of the kinetochore network in eukaryotes as revealed by comparative genomics
Source: EMBO Rep. 2017 Jun 22;18(9):1559–71. doi: 10.15252/embr.201744102 (PMC5579357; doi:10.15252/embr.201744102)
Supplement: Supplementary file 1 — Appendix [file EMBR-18-1559-s001.pdf]

Appendix for van Hooff et al., **The kinetochore network in eukaryotes as revealed by comparative genomics** (2017)

Table of contents

1. Appendix Table S1 2
2. Appendix Table S2 3
3. Appendix Table S3 9
4. Appendix Figure S1 11
5. Appendix Figure S2 12
6. Appendix Figure S3 13
7. Appendix References 14

Appendix Table S1: Measures of protein diversity in the set of kinetochore and APC/C proteins. Scores present the average across the proteins, except in the case of completeness (average across species). Statistical validity was assessed by performing unpaired, two-sided t-test.

Diversity feature (p-value kinetochore vs. APC/C)	Kinetochore (average)	APC/C (average)
Frequency (p=0.0045)	0.460	0.689
Entropy (p=0.0248)	0.731	0.578
Pearson correlation coefficient (p=0.0006)	0.219	0.267
Completeness (p=3.66e-13)	0.481	0.701
Loss (Dollo parsimony, p=0.140)	16.5	13.1
Transitions (p=0.636)	0.172	0.184
% Identity (human-mouse, p=0.0016)	74.8%	89.2%
dN/dS (human-mouse, p=1.80e-5)	0.245	0.059

Appendix Table S2: Sources of proteomes

Abbreviation*	Scientific name	Taxonomy ID	Download (m/d/y)	Assembly version	Reference/source
AANO	Aureococcus anophagefferens CCMP1984	44056	6/13/2013	v.1.0 (September 27, 2007)	[1]
ACAS	Acanthamoebe castellanii str. Neff	1257118	6/12/2013	Acastellanii.strNEFF v1 (January 9, 2013)	[2]
ACOE	Aquilegia coerulea Goldsmith	218851	17/7/2013	Release of the initial 8X unmapped Aquilegia coerulea Goldsmith genome	Aquilegia coerulea Genome Sequencing Project, http://phytozome.jgi.doe.gov/
AGAM	Anopheles gambiae str. PEST	180454	7/1/2013	AgamP3.7	[3]
AKER	Aplanochytrium kerguelense PBS07	702273	4/11/2013	V1	These sequence data were produced by the US Department of Energy Joint Genome Institute http://www.jgi.doe.gov/ in collaboration with the user community.
ALAI	Albugo laibachii Nc14	890382	7/1/2013	ENA 1 (2011-08-ENA)	[4]
ALIM	Aurantiochytrium limacinum ATCC MYA-1381	717989	4/11/2013	V1	These sequence data were produced by the US Department of Energy Joint Genome Institute http://www.jgi.doe.gov/ in collaboration with the user community.
AMAC	Allomyces macrogynus ATCC 38327	578462	6/10/2013	V1, March 1, 2012	Origins of Multicellularity Sequencing Project, Broad Institute of Harvard and MIT (http://www.broadinstitute.org/)
AQUE	Amphimedon queenslandica	400682	6/12/2013	V1.0, (May 28, 2010)	[5]
ATHA	Arabidopsis thaliana	3702	7/3/2013	TAIR10, November 17, 2010	[6]
ATRI	Amborella trichopoda	13333	5/1/2013	Unknown	[7]
BDEN	Batrachochytrium dendrobatidis JAM81	684364	6/3/2013	V1	These sequence data were produced by the US Department of Energy Joint Genome Institute http://www.jgi.doe.gov/ in collaboration with the user community
BFLO	Branchiostoma floridae	7739	7/1/2013	Unknown	[8]
BHOM	Blastocystis hominis	12968	6/13/2013	V1	[9]

BMAL	Brugia malayi	6279	6/27/2013	WS238	[10,11]
BNAT	Bigelowiella natans CCMP2755	753081	6/13/2013	V1	[12]
BPRA	Bathycoccus prasinos RCC1005	1075084	5/30/2013	V1	[13]
CANG	Catenaria anguillulae PL171	765915	6/10/2013	V1	These sequence data were produced by the US Department of Energy Joint Genome Institute http://www.jgi.doe.gov/ in collaboration with the user community.
CCOR	Conidiobolus coronatus NRRL28638	796925	6/10/2013	V1	[14]
CELE	Caenorhabditis elegans	6239	7/1/2013	WBcel235	[15]
CGLA	Candida glabrata CBS138	284593	6/13/2013	7/12/2012	[16,17]
CINT	Ciona intestinalis	7719	6/27/2013	KH (April 29, 2011)	[18]
CMER	Cyanidioschyzon merolae 10D	280699	6/13/2013	V1	[19]
CNEO	Cryptococcus neoformans	235443	6/12/2013	V4	Cryptococcus neoformans var. grubii H99 Sequencing Project, Broad Institute of Harvard and MIT (http://www.broadinstitute.org/)[20]
COWC	Capsaspora owczarzaki ATCC 30864	595528	5/31/2013	V2	Origins of Multicellularity Sequencing Project, Broad Institute of Harvard and MIT (http://www.broadinstitute.org/)
CPAI	Cryptosporidium parvum Iowa II	414452	5/29/2013	2/23/2007	[21]
CPAR	Cyanophora paradoxa	2762	6/13/2013	V1, November 2010	[22]
CREI	Chlamydomonas reinhardtii	3055	7/2/2013	Unknown	[23]
CREV	Coemansia reversa NRRL 1564	763665	6/10/2013	V1	[14]
CSUB	Coccomyxa subellipsoidea C-169	574566	6/14/2013	V2.0 (April 13, 2012)	[24]
CVAR	Chlorella variabilis NC64A	554065	5/30/2013	V1	[25]
DDIS	Dictyostelium discoideum AX4	352472	5/30/2013	Unknown	[26]

DHAN	Debaryomyces hansenii CBS767	284592	6/12/2013	V2, February 9, 2012	[16]
DMEL	Drosophila melanogaster	7227	6/26/2013	5.51, May 7, 2013	[27,28]
DRER	Danio rerio	7955	7/1/2013	Zv9	[29]
EAED	Edhazardia aedis USNM 41457	1003232	5/27/2013	4/30/2013	Microsporidia Comparative Sequencing Project, Broad Institute of Harvard and MIT (http://www.broadinstitute.org/)
EHIS	Entamoeba histolytica HM-1:IMSS	294381	5/31/2013	11/27/2009	[30]
EHUX	Emiliania huxleyi CCMP1516	280463	6/26/2013	V1	[31]
EINT	Encephalitozoon intestinalis ATCC 50506	876142	5/31/2013	10/18/2010	[32]
ESIL	Ectocarpus siliculosus	2880	6/13/2013	Unknown	[33]
GINT	Giardia intestinalis assemblage A	941442	5/29/2013	2/8/2013	[34]
GSUL	Galdieria sulphuraria	130081	6/13/2013	ASM34128v1 (February 25, 2013)	[35]
GTHE	Guillardia theta CCMP2712	905079	7/1/2013	Guith1 (2012-12-EnsemblProtist)	[12]
HPAR	Hyaloperonospora parasitica	272952	7/5/2013	Unknown	Saprolegnia genome Sequencing Project, Broad Institute of Harvard and MIT (http://www.broadinstitute.org/)
HSAP	Homo sapiens	9606	7/1/2013	GRCh37.p10	[36]
KLAC	Kluyveromyces lactis NRRL Y-1140	284590	6/12/2013	ASM251v1, (July 2, 2004)	[16]
LMAJ	Leishmania major strain Friedlin	347515	5/29/2013	10/20/2010	[37]
MBRE	Monosiga brevicollis MX1 / ATCC 50154	431895 / 487148	6/12/2013	V1.0, (December 20, 2007)	[38]
MCIR	Mucor circinelloides 1006PhL	1220926	6/28/2013	V1	[39]
MELO	Mortierella elongata	310910	6/10/2013	V1	These sequence data were produced by the US Department of Energy Joint Genome Institute http://www.jgi.doe.gov/ in collaboration with the user community.

MLEI	Mnemiopsis leidyi	27923	5/29/2013	Initial release	http://research.nhgri.nih.gov/mnemiopsis/
MMUS	Mus musculus	10090	7/1/2013	GRCm38.p1	[40]
MSPE	Micromonas species RCC299	296587	6/17/2013	ASM9098v1 (April 10, 2009)	[41]
MVER	Mortierella verticillata NRRL 6337	1069443	6/10/2013	2/17/2011	Origins of Multicellularity Sequencing Project, Broad Institute of Harvard and MIT (http://www.broadinstitute.org/)
NCRA	Neurospora crassa OR74A	367110	6/12/2013	3/11/2013	Neurospora crassa Sequencing Project, Broad Institute of Harvard and MIT (http://www.broadinstitute.org/)[42]
NGAD	Nannochloropsis gaditana CCMP526	1093141	6/13/2013	1.1	[43]
NGRU	Naegleria gruberi strain NEG-M	744533	6/19/2013	1	[44]
NVEC	Nematostella vectensis	45351	5/29/2013	1	[45]
OLUC	Ostreococcus lucimarinus CCE9901	436017	7/3/2013	Unknown	[46]
OSAT	Oryza sativa japonica	39947	7/1/2013	MSU6 (2009-01-MSU6)	[47]
OTRI	Oxytricha trifallax	1172189	5/29/2013	2/12/2012	[48]
PBLA	Phycomyces blakesleeanus NRRL1555	763407	6/10/2013	V2	These sequence data were produced by the US Department of Energy Joint Genome Institute http://www.jgi.doe.gov/ in collaboration with the user community.
PFAL	Plasmodium falciparum 3D7	36329	5/29/2013	3	[49]
PINF	Phytophthora infestans T30-4	403677	6/13/2013	ASM14294v1, (June 17, 2009)	Phytophthora infestans Sequencing Project, Broad Institute of Harvard and MIT (http://www.broadinstitute.org/)[50]
PMAR	Perkinsus marinus ATCC 50983	423536	5/29/2013	Unknown	
PPAL	Polysphondylium pallidum PN500	670386	5/31/2013	PolPal_Dec2009, (January 29, 2010)	[51]
PPAT	Physcomitrella patens subsp. patens	145481	7/4/2013	V1.6	[52,53]
PTET	Paramecium tetraurelia	5888	5/29/2013	v1.78	[54]

PTRI	Phaeodactylum tricornutum CCAP1055/1	556484	6/13/2013	ASM15095v2 (December 12, 2008)	[55]
SCER	Saccharomyces cerevisiae S288C	559292	6/13/2013	Release R64-1-1	[56]
SKOW	Saccoglossus kowalevskii	10224	7/1/2013	Unknown	[57]
SMAN	Schistosoma mansoni	6183	5/29/2013	5	[58]
SMIN	Symbiodinium minutum	1202447	17/7/13	v1.0	[59]
SMOE	Selaginella moellendorffii	88036	7/1/2013	v1.0 (2011-05-ENA)	[60]
SPOM	Schizosaccharomyces pombe (strain 972 / ATCC 24843)	4896	7/1/2013	ASM294v1 (2012-03-PomBase)	[61]
SPUN	Spizellomyces punctatus DAOM BR117	645134	6/3/2013 / 6/11/2013	3/1/2012	Origins of Multicellularity Sequencing Project, Broad Institute of Harvard and MIT (http://www.broadinstitute.org/)
SROS	Salpingoeca rosetta	946362	5/31/2013	V1	Origins of Multicellularity Sequencing Project, Broad Institute of Harvard and MIT (http://www.broadinstitute.org/)[62]
TADH	Trichoplax adhaerens	10228	6/12/2013	V1.0, (June 17, 2008)	[63]
TBRU	Trypanosoma brucei TREU 927	185431	6/19/2013	2013-01-16	[64]
TGON	Toxoplasma gondii ME49	508771	5/29/2013	7/23/2012	Lis Caler, J. Craig Venter Institute
TPSE	Thalassiosira pseudonana	296543	6/13/2013	ASM14940v1 (January 16, 2009)	[65]
TRUB	Takifugu rubripes	31033	7/1/2013	FUGU5 (October 13, 2011)	[66]
TTHE	Tetrahymena thermophila	5911	5/29/2013	oct2008	[67,68]
TTRA	Thecamonas trahens ATCC 50062	461836	6/13/2013	10/28/2011	Origins of Multicellularity Sequencing Project, Broad Institute of Harvard and MIT (http://www.broadinstitute.org/)
TVAG	Trichomonas vaginalis G3	412133	5/29/2013	1/11/2007	[69]
UMAY	Ustilago maydis	237631	6/12/2013	ASM32847v1 (March 5, 2007)	[70]

VCAR	Volvox carteri	3068	7/3/2013	Unknown	[71]
VCUL	Vavraia culicis floridensis	948595	5/27/2013	4/30/2013	Microsporidia Comparative Sequencing Project, Broad Institute of Harvard and MIT (http://www.broadinstitute.org/)
XTRO	Xenopus tropicalis	8364	7/1/2013	Xtropicalis_v7 (December 19, 2012)	[72]
YLIP	Yarrowia lipolytica CLIB 122	284591	7/1/2013	ASM252v1 (2012-05-EnsemblFungi)	[16]

Appendix Table S3: Phylogenetic profiles of the kinetochore proteins were compared to (sub)families defined using PANTHER10 [73]. The PANTHER10 (sub)families that have similar phylogenetic profiles as many kinetochore proteins (as measured by the Pearson correlation coefficient, indicated by their frequency in the top 30 of each kinetochore protein) are shown here.

PANTHER10 (sub)family	Frequency (top 30)	Pearson correlation coefficient (r, average)	Protein	Information from
PTHR11444.SF3|ARGININOSUCCINATE LYASE	12	0.4914568	ARGININOSUCCINATE LYASE	human
PTHR28080|FAMILY NOT NAMED	12	0.485799234	Pex3:Peroxisomal Biogenesis Factor 3	human
PTHR12309.SF12|CENTROMERE PROTEIN N	12	0.770296195	CenpN	human
PTHR14582|FAMILY NOT NAMED	11	0.682881415	CenpO	human
PTHR23342.SF0|N-ACETYLGLUTAMATE SYNTHASE, MITOCHONDRIAL	11	0.620553285	NAGS:N-Acetylglutamate Synthase	human
PTHR28262|FAMILY NOT NAMED	10	0.773155189	Spc19	human
PTHR12856|TRANSCRIPTION INITIATION FACTOR IIH-RELATED	10	0.567686548	GTF2H1:General Transcription Factor IIH Subunit 1	human
PTHR14401|FAMILY NOT NAMED	10	0.714825979	CenpK	human
PTHR10606.SF39|6-PHOSPHOFRUCTO-2-KINASE/FRUCTOSE-2,6-BISPHOSPHATASE YLR345W-RELATED	10	0.773155189	Similar to 6-phosphofructo-2-kinase enzymes	yeast
PTHR14778|FAMILY NOT NAMED	10	0.642404326	Dsn1	human
PTHR31749|FAMILY NOT NAMED	10	0.6242374	Nsl1	human
PTHR18460|UNCHARACTERIZED	10	0.543702758	TTI1: TELO2 Interacting Protein 1	human
PTHR34832|FAMILY NOT NAMED	10	0.635973745	CenpW	human
PTHR10555.SF136|VACUOLAR PROTEIN SORTING-ASSOCIATED PROTEIN 17	10	0.773155189	Vps17	yeast
PTHR28017|FAMILY NOT NAMED	10	0.773155189	Dad3	yeast
**PTHR21286|NUCLEAR PORE COMPLEX PROTEIN NUP160**	**10**	**0.533128741**	**Nup160**	**human**
PTHR31382.SF4|NA(+)/H(+) ANTIPORTER	9	0.764295654	NHA1: Na+/H+ antiporter	yeast
PTHR24343.SF137|SERINE/THREONINE-PROTEIN KINASE RTK1-RELATED	9	0.76076875	RTK1	yeast

PTHR11689.SF93|ANION/PROTON EXCHANGE TRANSPORTER GEF1	9	0.76076875	Gef1	yeast
PTHR11266.SF8|MPV17-LIKE PROTEIN 2	9	0.592282748	MPV17L2:Mitochondrial Inner Membrane Protein Like 2	human
PTHR31740.SF2|CENTROMERE PROTEIN L	9	0.667189989	CenpL	human
PTHR28051.SF1|RESISTANCE TO GLUCOSE REPRESSION PROTEIN 1	9	0.709057063	REG1:Regulatory subunit of type 1 protein phosphatase Glc7p	yeast
PTHR28113|FAMILY NOT NAMED	9	0.799278566	Dam1	yeast
PTHR23139.SF60|CENTROMERE PROTEIN I	9	0.661327982	CenpI	human
PTHR12064.SF29|PROTEIN MAM3	9	0.756454928	Mam3	yeast
PTHR28036|FAMILY NOT NAMED	9	0.787260404	Dad2	yeast
PTHR23168|MITOTIC SPINDLE ASSEMBLY CHECKPOINT PROTEIN MAD1 MITOTIC ARREST DEFICIENT-LIKE PROTEIN 1	9	0.550060967	Mad1	human
PTHR28662|FAMILY NOT NAMED	9	0.654806603	CenpH	yeast
PTHR28077|FAMILY NOT NAMED	9	0.731332783	Kei1:Kex2-cleavable protein Essential for Inositol phosphorylceramide synthesis	yeast
PTHR11365.SF11|SUBFAMILY NOT NAMED	9	0.731332783	OXP1:OxoProlinase	yeast

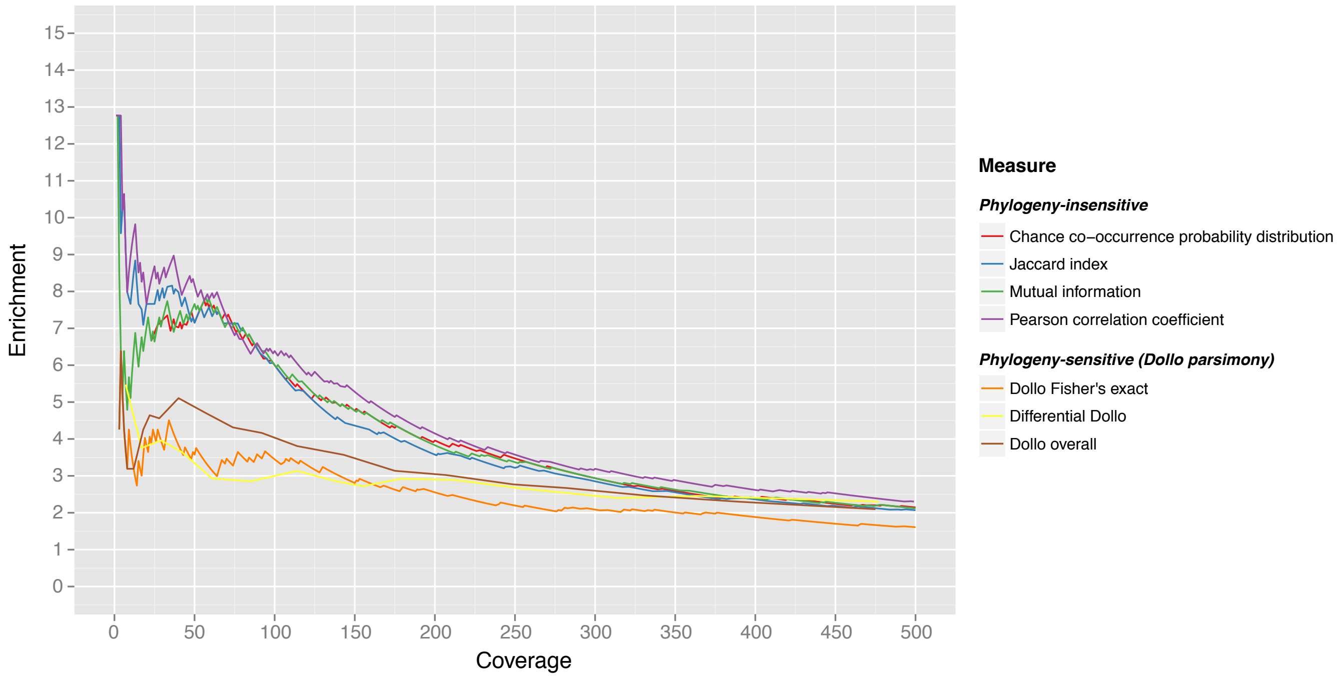

Appendix Figure S1. Performance of various measures that compare phylogenetic profiles in predicting physically interacting proteins

Various metrics quantify the similarity between phylogenetic profiles, such as Pearson correlation coefficient, hamming distance, chance co-occurrence probability distribution, jaccard index, mutual information [74,75] and various phylogeny-sensitive measures such as those based on Dollo parsimony [76]. We compared these metrics by assessing how well they return known physically interacting genes. A set of physically interacting proteins was obtained for our proteins of interest (kinetochore proteins) using the BioGRID [77]. For each metric, we calculated the enrichment of these confirmed interacting protein pairs among pairs having a given phylogenetic profile similarity score (converted into the coverage of all possible protein pairs at that similarity score). Across most scores, the Pearson correlation coefficient returns the highest number of interacting pairs. For this Pearson correlation coefficient (r), the threshold t was set at the r value that yields 6-fold enrichment of interacting pairs relative to pairs for which no interaction is observed (r=0.477).

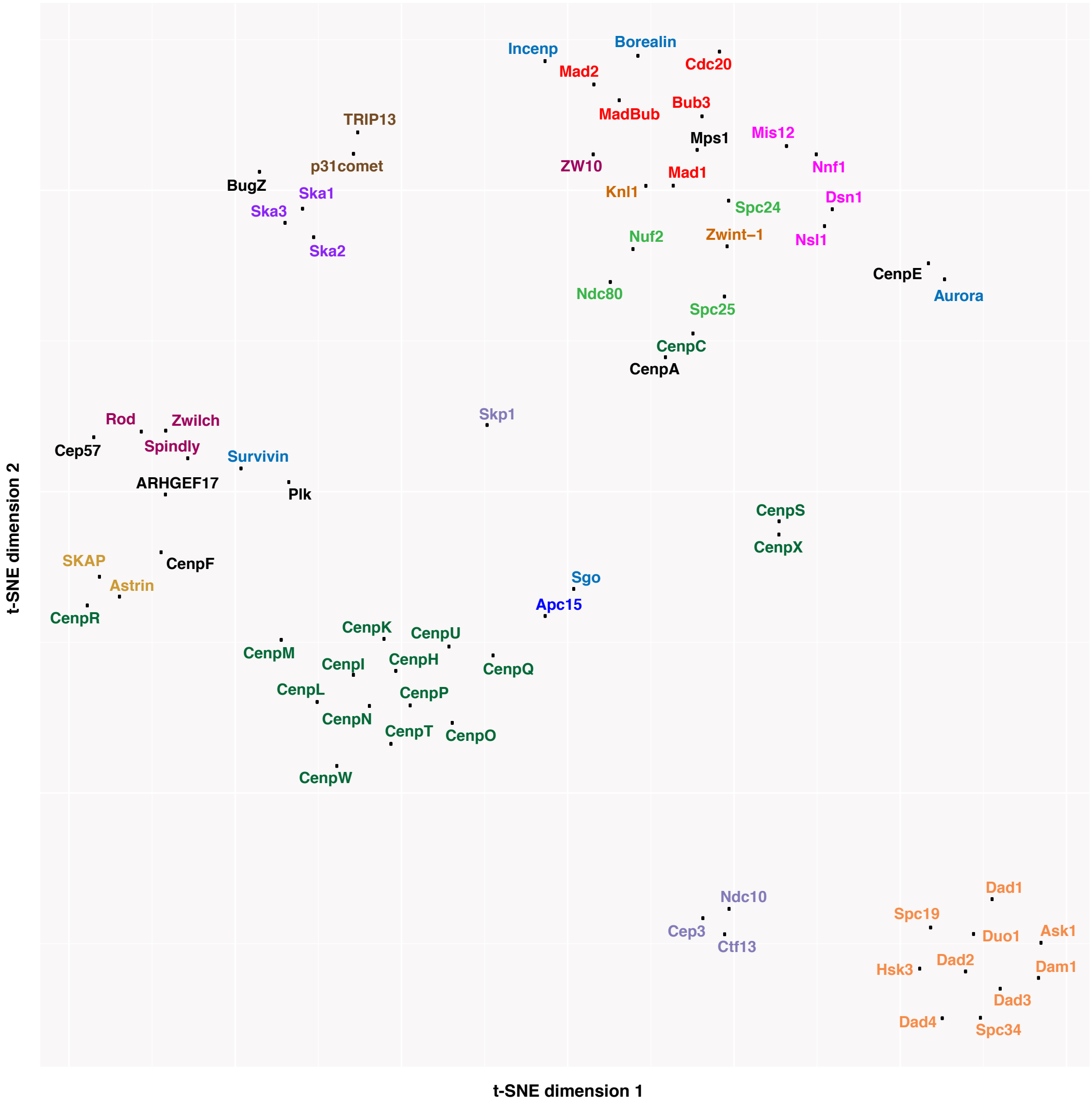

Appendix Figure S2. T-SNE map of kinetochore proteins

The kinetochore proteins were visualized using a Barnes-Hut implementation of t-Distributed Stochastic Neighbor Embedding (t-SNE) [78] based on their pairwise distances measured by the Pearson correlation coefficient of the phylogenetic profiles. The protein names are colored according to their complex memberships, identical to Figure 1.

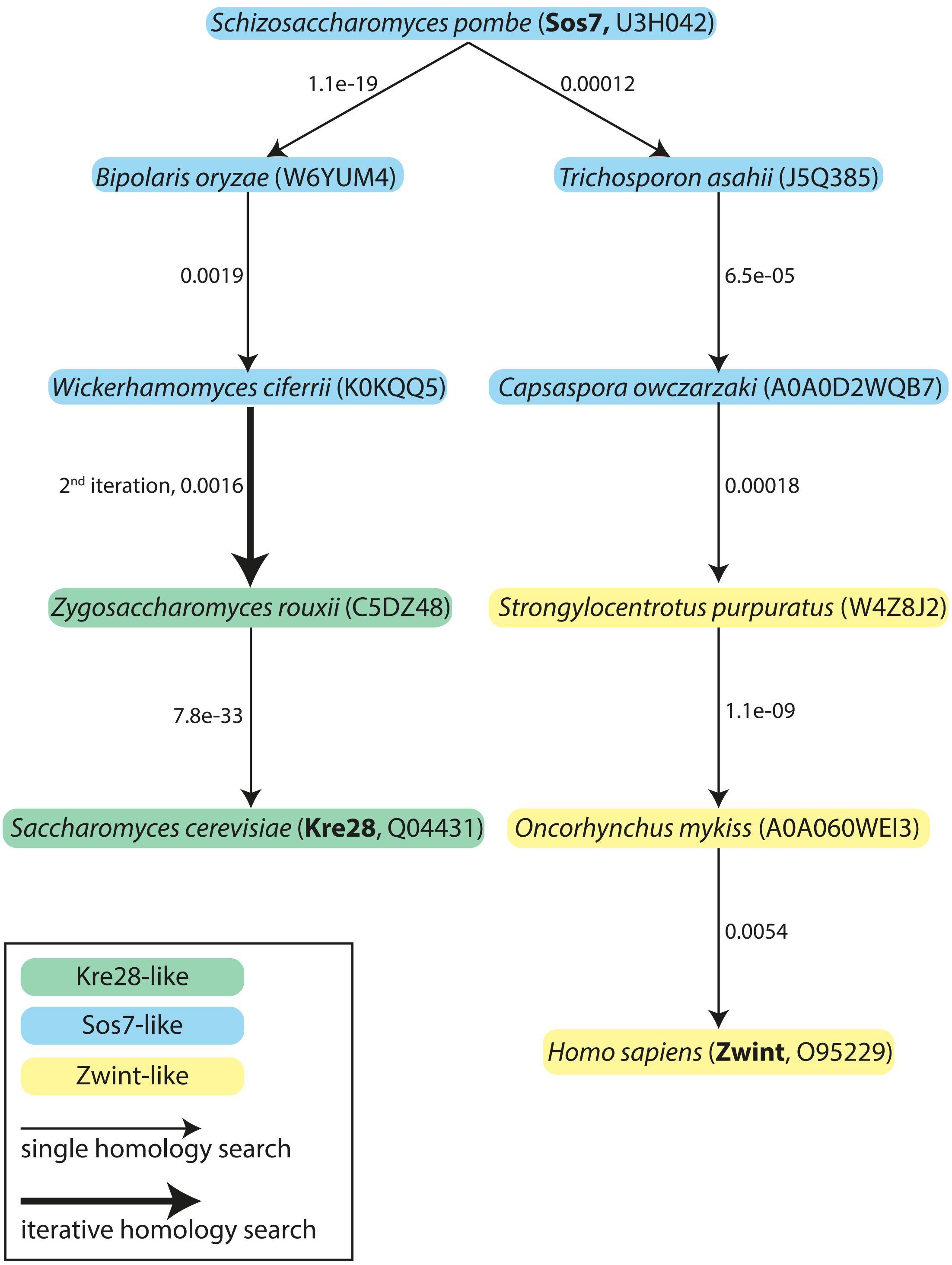

Appendix Figure S3. Establishing homology between Zwint-1, Sos7 and Kre28
Sequences that link Sos7 (Schizosaccharomyces pombe), Kre28 (Saccharomyces cerevisiae) and Zwint-1 (Homo sapiens) with homology searches (arrows) and corresponding E-values (searches against UniProtKB database performed online on October 1 2015, http://www.ebi.ac.uk/Tools/hmmer/), indicated by species and UniProt IDs. Colors represent to which protein that sequence is most similar. Of the sequences indicated here, in addition to Sos7, Kre28 and Zwint-1 also the hit in Capsaspora owczarzaki is in the proteome database used in this study. We used sequences in additional species to connect sequences in the database, as indicated by this scheme.

Appendix References

1. Gobler CJ, Berry DL, Dyhrman ST, Wilhelm SW, Salamov A, Lobanov AV, Zhang Y, Collier JL, Wurch LL, Kustka AB*, et al.* (2011) Niche of harmful alga Aureococcus anophagefferens revealed through ecogenomics. *Proceedings of the National Academy of Sciences of the United States of America* **108:** 4352-4357
2. Clarke M, Lohan AJ, Liu B, Lagkouvardos I, Roy S, Zafar N, Bertelli C, Schilde C, Kianianmomeni A, Burglin TR*, et al.* (2013) Genome of Acanthamoeba castellanii highlights extensive lateral gene transfer and early evolution of tyrosine kinase signaling. *Genome biology* **14:** R11
3. Holt RA, Subramanian GM, Halpern A, Sutton GG, Charlab R, Nusskern DR, Wincker P, Clark AG, Ribeiro JM, Wides R*, et al.* (2002) The genome sequence of the malaria mosquito Anopheles gambiae. *Science* **298:** 129-149
4. Kemen E, Gardiner A, Schultz-Larsen T, Kemen AC, Balmuth AL, Robert-Seilaniantz A, Bailey K, Holub E, Studholme DJ, Maclean D*, et al.* (2011) Gene gain and loss during evolution of obligate parasitism in the white rust pathogen of Arabidopsis thaliana. *PLoS biology* **9:** e1001094
5. Srivastava M, Simakov O, Chapman J, Fahey B, Gauthier ME, Mitros T, Richards GS, Conaco C, Dacre M, Hellsten U*, et al.* (2010) The Amphimedon queenslandica genome and the evolution of animal complexity. *Nature* **466:** 720-726
6. (2000) Analysis of the genome sequence of the flowering plant Arabidopsis thaliana. *Nature* **408:** 796-815
7. (2013) The Amborella genome and the evolution of flowering plants. *Science* **342:** 1241089
8. Putnam NH, Butts T, Ferrier DE, Furlong RF, Hellsten U, Kawashima T, Robinson-Rechavi M, Shoguchi E, Terry A, Yu JK*, et al.* (2008) The amphioxus genome and the evolution of the chordate karyotype. *Nature* **453:** 1064-1071
9. Denoeud F, Roussel M, Noel B, Wawrzyniak I, Da Silva C, Diogon M, Viscogliosi E, Brochier-Armanet C, Couloux A, Poulain J*, et al.* (2011) Genome sequence of the stramenopile Blastocystis, a human anaerobic parasite. *Genome biology* **12:** R29
10. Ghedin E, Wang S, Spiro D, Caler E, Zhao Q, Crabtree J, Allen JE, Delcher AL, Guiliano DB, Miranda-Saavedra D*, et al.* (2007) Draft genome of the filarial nematode parasite Brugia malayi. *Science* **317:** 1756-1760
11. Yook K, Harris TW, Bieri T, Cabunoc A, Chan J, Chen WJ, Davis P, de la Cruz N, Duong A, Fang R*, et al.* (2012) WormBase 2012: more genomes, more data, new website. *Nucleic acids research* **40:** D735-741
12. Curtis BA, Tanifuji G, Burki F, Gruber A, Irimia M, Maruyama S, Arias MC, Ball SG, Gile GH, Hirakawa Y*, et al.* (2012) Algal genomes reveal evolutionary mosaicism and the fate of nucleomorphs. *Nature* **492:** 59-65
13. Moreau H, Verhelst B, Couloux A, Derelle E, Rombauts S, Grimsley N, Van Bel M, Poulain J, Katinka M, Hohmann-Marriott MF*, et al.* (2012) Gene functionalities and genome structure in Bathycoccus prasinos reflect cellular specializations at the base of the green lineage. *Genome biology* **13:** R74
14. Chang Y, Wang S, Sekimoto S, Aerts AL, Choi C, Clum A, LaButti KM, Lindquist EA, Yee Ngan C, Ohm RA*, et al.* (2015) Phylogenomic Analyses Indicate that Early Fungi Evolved Digesting Cell Walls of Algal Ancestors of Land Plants. *Genome Biol Evol* **7:** 1590-1601
15. (1998) Genome sequence of the nematode C. elegans: a platform for investigating biology. *Science* **282:** 2012-2018

16. Dujon B, Sherman D, Fischer G, Durrens P, Casaregola S, Lafontaine I, De Montigny J, Marck C, Neuveglise C, Talla E*, et al.* (2004) Genome evolution in yeasts. *Nature* **430:** 35-44
17. Inglis DO, Arnaud MB, Binkley J, Shah P, Skrzypek MS, Wymore F, Binkley G, Miyasato SR, Simison M, Sherlock G (2012) The Candida genome database incorporates multiple Candida species: multispecies search and analysis tools with curated gene and protein information for Candida albicans and Candida glabrata. *Nucleic acids research* **40:** D667-674
18. Dehal P, Satou Y, Campbell RK, Chapman J, Degnan B, De Tomaso A, Davidson B, Di Gregorio A, Gelpke M, Goodstein DM*, et al.* (2002) The draft genome of Ciona intestinalis: insights into chordate and vertebrate origins. *Science* **298:** 2157-2167
19. Matsuzaki M, Misumi O, Shin IT, Maruyama S, Takahara M, Miyagishima SY, Mori T, Nishida K, Yagisawa F, Nishida K*, et al.* (2004) Genome sequence of the ultrasmall unicellular red alga Cyanidioschyzon merolae 10D. *Nature* **428:** 653-657
20. Loftus BJ, Fung E, Roncaglia P, Rowley D, Amedeo P, Bruno D, Vamathevan J, Miranda M, Anderson IJ, Fraser JA*, et al.* (2005) The genome of the basidiomycetous yeast and human pathogen Cryptococcus neoformans. *Science* **307:** 1321-1324
21. Abrahamsen MS, Templeton TJ, Enomoto S, Abrahante JE, Zhu G, Lancto CA, Deng M, Liu C, Widmer G, Tzipori S*, et al.* (2004) Complete genome sequence of the apicomplexan, Cryptosporidium parvum. *Science* **304:** 441-445
22. Price DC, Chan CX, Yoon HS, Yang EC, Qiu H, Weber AP, Schwacke R, Gross J, Blouin NA, Lane C*, et al.* (2012) Cyanophora paradoxa genome elucidates origin of photosynthesis in algae and plants. *Science* **335:** 843-847
23. Merchant SS, Prochnik SE, Vallon O, Harris EH, Karpowicz SJ, Witman GB, Terry A, Salamov A, Fritz-Laylin LK, Marechal-Drouard L*, et al.* (2007) The Chlamydomonas genome reveals the evolution of key animal and plant functions. *Science* **318:** 245-250
24. Blanc G, Agarkova I, Grimwood J, Kuo A, Brueggeman A, Dunigan DD, Gurnon J, Ladunga I, Lindquist E, Lucas S*, et al.* (2012) The genome of the polar eukaryotic microalga Coccomyxa subellipsoidea reveals traits of cold adaptation. *Genome biology* **13:** R39
25. Blanc G, Duncan G, Agarkova I, Borodovsky M, Gurnon J, Kuo A, Lindquist E, Lucas S, Pangilinan J, Polle J*, et al.* (2010) The Chlorella variabilis NC64A genome reveals adaptation to photosymbiosis, coevolution with viruses, and cryptic sex. *The Plant cell* **22:** 2943-2955
26. Eichinger L, Pachebat JA, Glockner G, Rajandream MA, Sucgang R, Berriman M, Song J, Olsen R, Szafranski K, Xu Q*, et al.* (2005) The genome of the social amoeba Dictyostelium discoideum. *Nature* **435:** 43-57
27. Adams MD, Celniker SE, Holt RA, Evans CA, Gocayne JD, Amanatides PG, Scherer SE, Li PW, Hoskins RA, Galle RF*, et al.* (2000) The genome sequence of Drosophila melanogaster. *Science* **287:** 2185-2195
28. Attrill H, Falls K, Goodman JL, Millburn GH, Antonazzo G, Rey AJ, Marygold SJ (2015) FlyBase: establishing a Gene Group resource for Drosophila melanogaster. *Nucleic acids research*, 10.1093/nar/gkv1046
29. Howe K, Clark MD, Torroja CF, Torrance J, Berthelot C, Muffato M, Collins JE, Humphray S, McLaren K, Matthews L*, et al.* (2013) The zebrafish reference genome sequence and its relationship to the human genome. *Nature* **496:** 498-503
30. Loftus B, Anderson I, Davies R, Alsmark UC, Samuelson J, Amedeo P, Roncaglia P, Berriman M, Hirt RP, Mann BJ*, et al.* (2005) The genome of the protist parasite Entamoeba histolytica. *Nature* **433:** 865-868

31. Read BA, Kegel J, Klute MJ, Kuo A, Lefebvre SC, Maumus F, Mayer C, Miller J, Monier A, Salamov A*, et al.* (2013) Pan genome of the phytoplankton Emiliania underpins its global distribution. *Nature* **499:** 209-213
32. Corradi N, Pombert JF, Farinelli L, Didier ES, Keeling PJ (2010) The complete sequence of the smallest known nuclear genome from the microsporidian Encephalitozoon intestinalis. *Nature communications* **1:** 77
33. Cock JM, Sterck L, Rouze P, Scornet D, Allen AE, Amoutzias G, Anthouard V, Artiguenave F, Aury JM, Badger JH*, et al.* (2010) The Ectocarpus genome and the independent evolution of multicellularity in brown algae. *Nature* **465:** 617-621
34. Morrison HG, McArthur AG, Gillin FD, Aley SB, Adam RD, Olsen GJ, Best AA, Cande WZ, Chen F, Cipriano MJ*, et al.* (2007) Genomic minimalism in the early diverging intestinal parasite Giardia lamblia. *Science* **317:** 1921-1926
35. Schonknecht G, Chen WH, Ternes CM, Barbier GG, Shrestha RP, Stanke M, Brautigam A, Baker BJ, Banfield JF, Garavito RM*, et al.* (2013) Gene transfer from bacteria and archaea facilitated evolution of an extremophilic eukaryote. *Science* **339:** 1207-1210
36. Lander ES, Linton LM, Birren B, Nusbaum C, Zody MC, Baldwin J, Devon K, Dewar K, Doyle M, FitzHugh W*, et al.* (2001) Initial sequencing and analysis of the human genome. *Nature* **409:** 860-921
37. Ivens AC, Peacock CS, Worthey EA, Murphy L, Aggarwal G, Berriman M, Sisk E, Rajandream MA, Adlem E, Aert R*, et al.* (2005) The genome of the kinetoplastid parasite, Leishmania major. *Science* **309:** 436-442
38. King N, Westbrook MJ, Young SL, Kuo A, Abedin M, Chapman J, Fairclough S, Hellsten U, Isogai Y, Letunic I*, et al.* (2008) The genome of the choanoflagellate Monosiga brevicollis and the origin of metazoans. *Nature* **451:** 783-788
39. Lee SC, Billmyre RB, Li A, Carson S, Sykes SM, Huh EY, Mieczkowski P, Ko DC, Cuomo CA, Heitman J (2014) Analysis of a food-borne fungal pathogen outbreak: virulence and genome of a Mucor circinelloides isolate from yogurt. *mBio* **5:** e01390-01314
40. Waterston RH, Lindblad-Toh K, Birney E, Rogers J, Abril JF, Agarwal P, Agarwala R, Ainscough R, Alexandersson M, An P*, et al.* (2002) Initial sequencing and comparative analysis of the mouse genome. *Nature* **420:** 520-562
41. Worden AZ, Lee JH, Mock T, Rouze P, Simmons MP, Aerts AL, Allen AE, Cuvelier ML, Derelle E, Everett MV*, et al.* (2009) Green evolution and dynamic adaptations revealed by genomes of the marine picoeukaryotes Micromonas. *Science* **324:** 268-272
42. Galagan JE, Calvo SE, Borkovich KA, Selker EU, Read ND, Jaffe D, FitzHugh W, Ma LJ, Smirnov S, Purcell S*, et al.* (2003) The genome sequence of the filamentous fungus Neurospora crassa. *Nature* **422:** 859-868
43. Radakovits R, Jinkerson RE, Fuerstenberg SI, Tae H, Settlage RE, Boore JL, Posewitz MC (2012) Draft genome sequence and genetic transformation of the oleaginous alga Nannochloropis gaditana. *Nature communications* **3:** 686
44. Fritz-Laylin LK, Prochnik SE, Ginger ML, Dacks JB, Carpenter ML, Field MC, Kuo A, Paredez A, Chapman J, Pham J*, et al.* (2010) The genome of Naegleria gruberi illuminates early eukaryotic versatility. *Cell* **140:** 631-642
45. Putnam NH, Srivastava M, Hellsten U, Dirks B, Chapman J, Salamov A, Terry A, Shapiro H, Lindquist E, Kapitonov VV*, et al.* (2007) Sea anemone genome reveals ancestral eumetazoan gene repertoire and genomic organization. *Science* **317:** 86-94
46. Palenik B, Grimwood J, Aerts A, Rouze P, Salamov A, Putnam N, Dupont C, Jorgensen R, Derelle E, Rombauts S*, et al.* (2007) The tiny eukaryote Ostreococcus

provides genomic insights into the paradox of plankton speciation. *Proceedings of the National Academy of Sciences of the United States of America* **104:** 7705-7710
47. (2005) The map-based sequence of the rice genome. *Nature* **436:** 793-800
48. Swart EC, Bracht JR, Magrini V, Minx P, Chen X, Zhou Y, Khurana JS, Goldman AD, Nowacki M, Schotanus K*, et al.* (2013) The Oxytricha trifallax macronuclear genome: a complex eukaryotic genome with 16,000 tiny chromosomes. *PLoS biology* **11:** e1001473
49. Gardner MJ, Hall N, Fung E, White O, Berriman M, Hyman RW, Carlton JM, Pain A, Nelson KE, Bowman S*, et al.* (2002) Genome sequence of the human malaria parasite Plasmodium falciparum. *Nature* **419:** 498-511
50. Haas BJ, Kamoun S, Zody MC, Jiang RH, Handsaker RE, Cano LM, Grabherr M, Kodira CD, Raffaele S, Torto-Alalibo T*, et al.* (2009) Genome sequence and analysis of the Irish potato famine pathogen Phytophthora infestans. *Nature* **461:** 393-398
51. Heidel AJ, Lawal HM, Felder M, Schilde C, Helps NR, Tunggal B, Rivero F, John U, Schleicher M, Eichinger L*, et al.* (2011) Phylogeny-wide analysis of social amoeba genomes highlights ancient origins for complex intercellular communication. *Genome research* **21:** 1882-1891
52. Rensing SA, Lang D, Zimmer AD, Terry A, Salamov A, Shapiro H, Nishiyama T, Perroud PF, Lindquist EA, Kamisugi Y*, et al.* (2008) The Physcomitrella genome reveals evolutionary insights into the conquest of land by plants. *Science* **319:** 64-69
53. Zimmer AD, Lang D, Buchta K, Rombauts S, Nishiyama T, Hasebe M, Van de Peer Y, Rensing SA, Reski R (2013) Reannotation and extended community resources for the genome of the non-seed plant Physcomitrella patens provide insights into the evolution of plant gene structures and functions. *BMC genomics* **14:** 498
54. Arnaiz O, Sperling L (2011) ParameciumDB in 2011: new tools and new data for functional and comparative genomics of the model ciliate Paramecium tetraurelia. *Nucleic acids research* **39:** D632-636
55. Bowler C, Allen AE, Badger JH, Grimwood J, Jabbari K, Kuo A, Maheswari U, Martens C, Maumus F, Otillar RP*, et al.* (2008) The Phaeodactylum genome reveals the evolutionary history of diatom genomes. *Nature* **456:** 239-244
56. Goffeau A, Barrell BG, Bussey H, Davis RW, Dujon B, Feldmann H, Galibert F, Hoheisel JD, Jacq C, Johnston M*, et al.* (1996) Life with 6000 genes. *Science* **274:** 546, 563-547
57. Simakov O, Kawashima T, Marletaz F, Jenkins J, Koyanagi R, Mitros T, Hisata K, Bredeson J, Shoguchi E, Gyoja F*, et al.* (2015) Hemichordate genomes and deuterostome origins. *Nature* **527:** 459-465
58. Berriman M, Haas BJ, LoVerde PT, Wilson RA, Dillon GP, Cerqueira GC, Mashiyama ST, Al-Lazikani B, Andrade LF, Ashton PD*, et al.* (2009) The genome of the blood fluke Schistosoma mansoni. *Nature* **460:** 352-358
59. Shoguchi E, Shinzato C, Kawashima T, Gyoja F, Mungpakdee S, Koyanagi R, Takeuchi T, Hisata K, Tanaka M, Fujiwara M*, et al.* (2013) Draft assembly of the Symbiodinium minutum nuclear genome reveals dinoflagellate gene structure. *Current biology : CB* **23:** 1399-1408
60. Banks JA, Nishiyama T, Hasebe M, Bowman JL, Gribskov M, dePamphilis C, Albert VA, Aono N, Aoyama T, Ambrose BA*, et al.* (2011) The Selaginella genome identifies genetic changes associated with the evolution of vascular plants. *Science* **332:** 960-963
61. Wood V, Gwilliam R, Rajandream MA, Lyne M, Lyne R, Stewart A, Sgouros J, Peat N, Hayles J, Baker S*, et al.* (2002) The genome sequence of Schizosaccharomyces pombe. *Nature* **415:** 871-880

62. Fairclough SR, Chen Z, Kramer E, Zeng Q, Young S, Robertson HM, Begovic E, Richter DJ, Russ C, Westbrook MJ, *et al.* (2013) Premetazoan genome evolution and the regulation of cell differentiation in the choanoflagellate Salpingoeca rosetta. *Genome biology* **14:** R15
63. Srivastava M, Begovic E, Chapman J, Putnam NH, Hellsten U, Kawashima T, Kuo A, Mitros T, Salamov A, Carpenter ML, *et al.* (2008) The Trichoplax genome and the nature of placozoans. *Nature* **454:** 955-960
64. Berriman M, Ghedin E, Hertz-Fowler C, Blandin G, Renauld H, Bartholomeu DC, Lennard NJ, Caler E, Hamlin NE, Haas B, *et al.* (2005) The genome of the African trypanosome Trypanosoma brucei. *Science* **309:** 416-422
65. Armbrust EV, Berges JA, Bowler C, Green BR, Martinez D, Putnam NH, Zhou S, Allen AE, Apt KE, Bechner M, *et al.* (2004) The genome of the diatom Thalassiosira pseudonana: ecology, evolution, and metabolism. *Science* **306:** 79-86
66. Aparicio S, Chapman J, Stupka E, Putnam N, Chia JM, Dehal P, Christoffels A, Rash S, Hoon S, Smit A, *et al.* (2002) Whole-genome shotgun assembly and analysis of the genome of Fugu rubripes. *Science* **297:** 1301-1310
67. Eisen JA, Coyne RS, Wu M, Wu D, Thiagarajan M, Wortman JR, Badger JH, Ren Q, Amedeo P, Jones KM, *et al.* (2006) Macronuclear genome sequence of the ciliate Tetrahymena thermophila, a model eukaryote. *PLoS biology* **4:** e286
68. Stover NA, Krieger CJ, Binkley G, Dong Q, Fisk DG, Nash R, Sethuraman A, Weng S, Cherry JM (2006) Tetrahymena Genome Database (TGD): a new genomic resource for Tetrahymena thermophila research. *Nucleic acids research* **34:** D500-503
69. Carlton JM, Hirt RP, Silva JC, Delcher AL, Schatz M, Zhao Q, Wortman JR, Bidwell SL, Alsmark UC, Besteiro S, *et al.* (2007) Draft genome sequence of the sexually transmitted pathogen Trichomonas vaginalis. *Science* **315:** 207-212
70. Kamper J, Kahmann R, Bolker M, Ma LJ, Brefort T, Saville BJ, Banuett F, Kronstad JW, Gold SE, Muller O, *et al.* (2006) Insights from the genome of the biotrophic fungal plant pathogen Ustilago maydis. *Nature* **444:** 97-101
71. Prochnik SE, Umen J, Nedelcu AM, Hallmann A, Miller SM, Nishii I, Ferris P, Kuo A, Mitros T, Fritz-Laylin LK, *et al.* (2010) Genomic analysis of organismal complexity in the multicellular green alga Volvox carteri. *Science* **329:** 223-226
72. Hellsten U, Harland RM, Gilchrist MJ, Hendrix D, Jurka J, Kapitonov V, Ovcharenko I, Putnam NH, Shu S, Taher L, *et al.* (2010) The genome of the Western clawed frog Xenopus tropicalis. *Science* **328:** 633-636
73. Mi H, Muruganujan A, Thomas PD (2013) PANTHER in 2013: modeling the evolution of gene function, and other gene attributes, in the context of phylogenetic trees. *Nucleic Acids Research* **41:** D377-D386
74. Wu J, Kasif S, DeLisi C (2003) Identification of functional links between genes using phylogenetic profiles. *Bioinformatics* **19:** 1524-1530
75. Glazko GV, Mushegian AR (2004) Detection of evolutionarily stable fragments of cellular pathways by hierarchical clustering of phyletic patterns. *Genome biology* **5:** R32
76. Kensche PR, van Noort V, Dutilh BE, Huynen MA (2008) Practical and theoretical advances in predicting the function of a protein by its phylogenetic distribution. *Journal of the Royal Society, Interface / the Royal Society* **5:** 151-170
77. Chatr-aryamontri A, Oughtred R, Boucher L, Rust J, Chang C, Kolas NK, O'Donnell L, Oster S, Theesfeld C, Sellam A, *et al.* (2017) The BioGRID interaction database: 2017 update. *Nucleic Acids Research* **45:** D369-D379
78. Maaten Lvd, Hinton G (2008) Visualizing data using t-SNE. *Journal of Machine Learning Research* **9:** 2579-2605
