# Supplementary material for: Evolutionary dynamics of the kinetochore network in eukaryotes as revealed by comparative genomics
Source: EMBO Rep. 2017 Jun 22;18(9):1559–71. doi: 10.15252/embr.201744102 (PMC5579357; doi:10.15252/embr.201744102)
Supplement: Supplementary file 2 — Expanded View Figures PDF [file EMBR-18-1559-s002.pdf]

Expanded View Figures

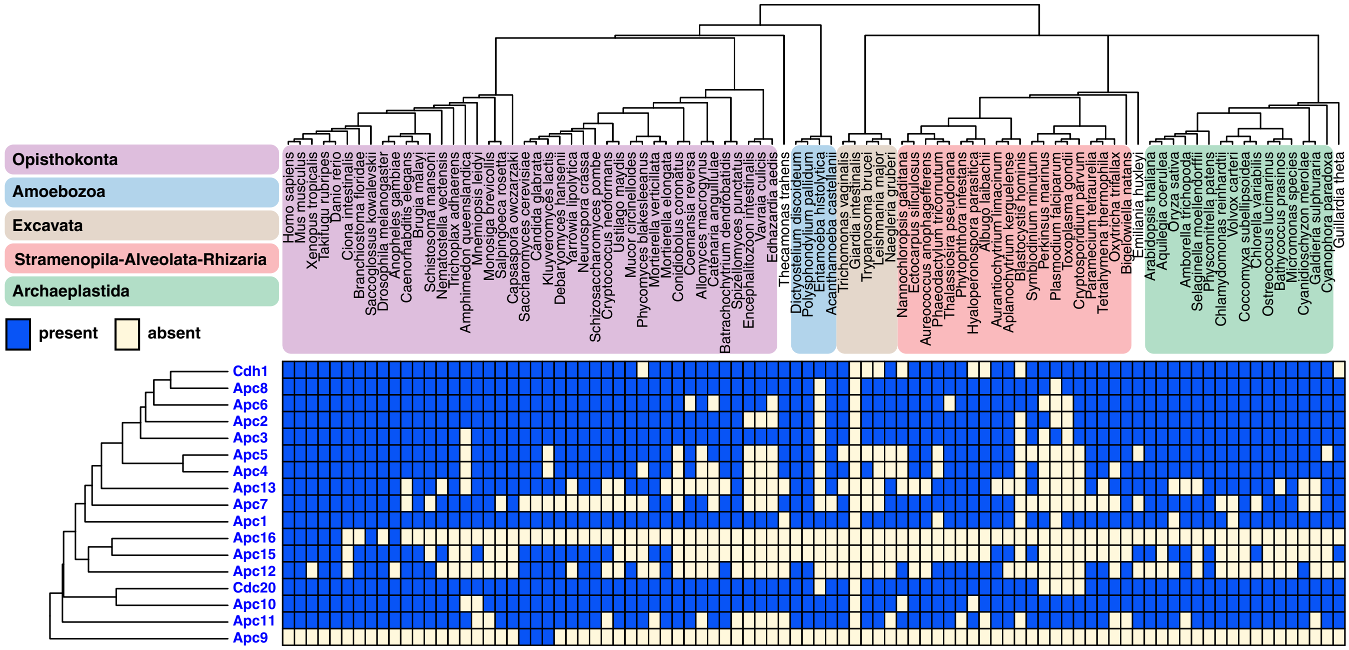

Figure EV1. Anaphase-promoting complex/cyclosome (APC/C) subunits across 90 eukaryotic lineages.

Presences and absences ("phylogenetic profiles") of APC/C subunits in 90 eukaryotic species. Top: Phylogenetic tree of the species in the genome set, with colored areas for the eukaryotic supergroups. Left side: APC/C proteins clustered by average linkage based on the pairwise Pearson correlation coefficients of their phylogenetic profiles. The orthologous sequences are available as fasta files in Dataset EV1, allowing full usage of our data for further evolutionary cell biology investigations.

 © 2017 The Authors

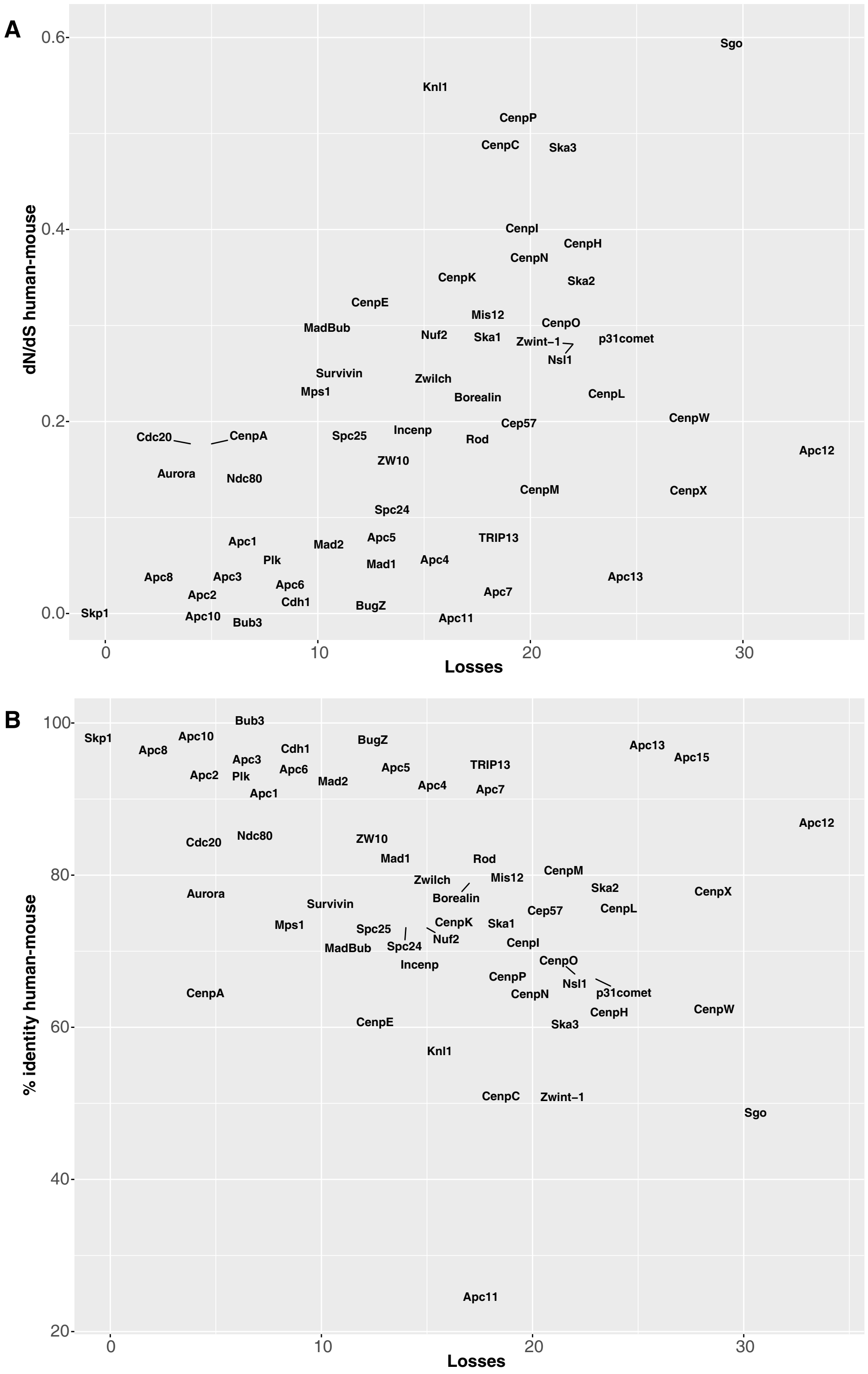

Figure EV2. Loss frequencies and sequence evolution of kinetochore and APC/C proteins.

A, B Scatter plots for loss frequencies and dN/dS values (A) and percent identity (B) of human–mouse orthologs for the kinetochore and APC/C proteins that were inferred to have been present in LECA. Loss frequencies and dN/dS values positively correlate (*P* = 3.9e-5, Spearman correlation), whereas loss frequencies and percent identity negatively correlate (*P* = 0.0005, Spearman correlation).

© 2017 The Authors

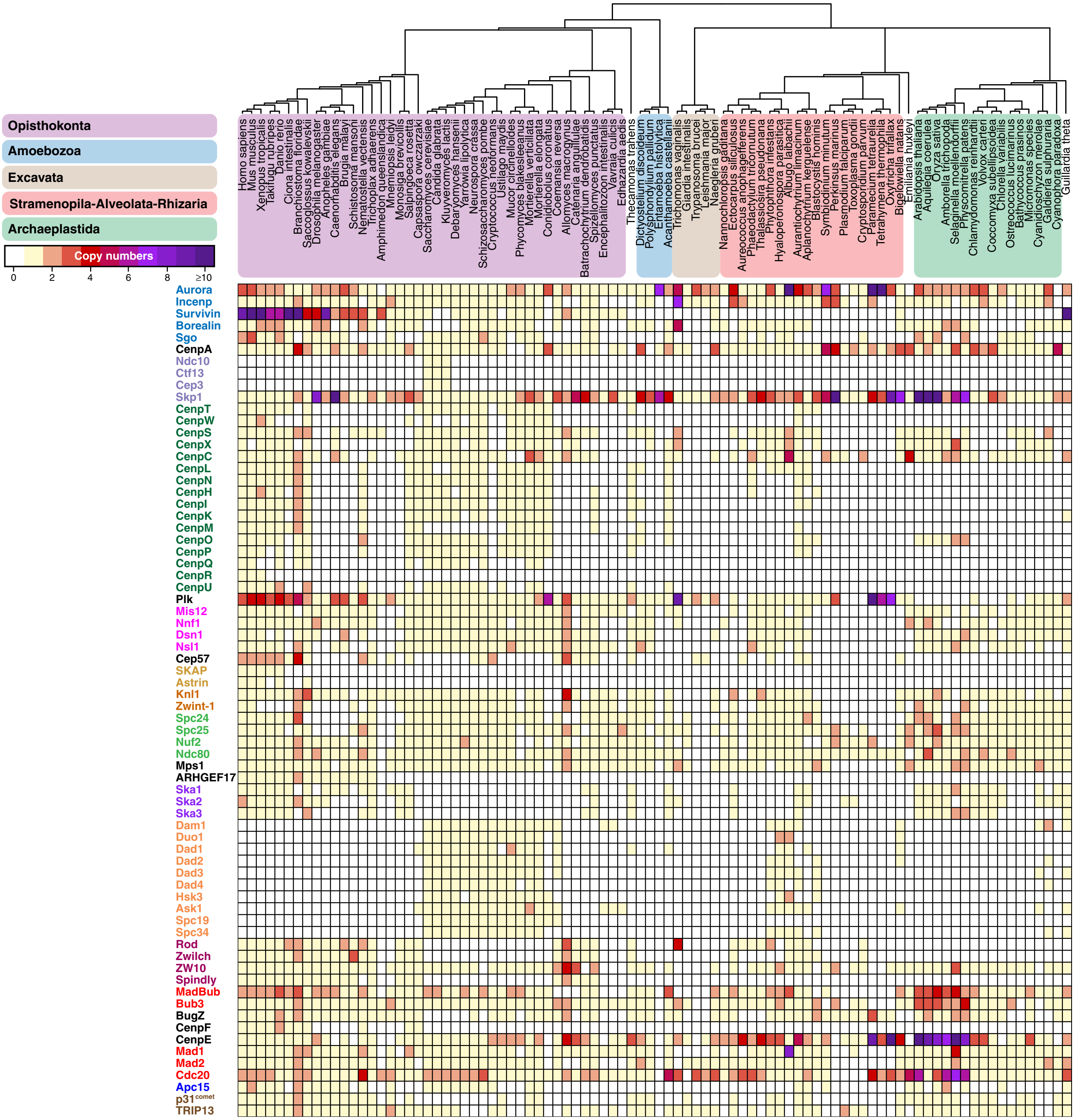

Figure EV3. Copy numbers of kinetochore proteins.

Heatmap indicating the copy numbers of each kinetochore protein in the 90 eukaryotic lineages. Please note that these copy numbers might contain some over- and underestimates due to unpredicted or imperfectly predicted genes and database errors.

 © 2017 The Authors

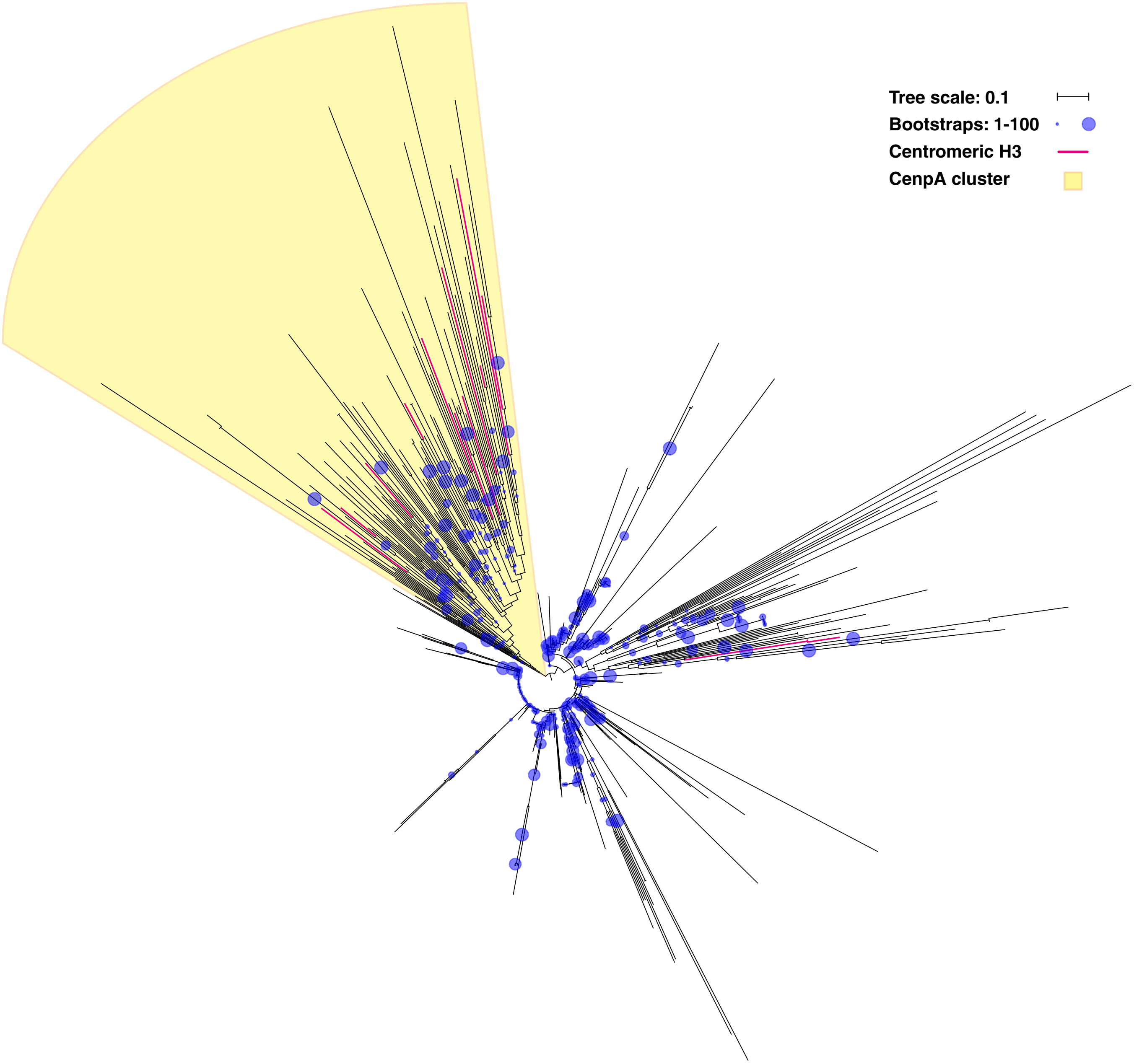

Figure EV4. Gene phylogeny of histone H3 homologs.

To find the putative orthologs of CenpA, we first aligned candidate orthologous sequences, which were experimentally identified centromeric H3 variants in divergent species (indicated with a pink branch in this phylogeny). From this alignment, we constructed a profile HMM and performed multiple HMM searches through our local proteome database. From these searches, we selected 831 sequences (belonging to the histone H3 family), aligned these and constructed the gene phylogeny, which is presented in this figure (see also Materials and Methods). We rooted the phylogeny on the cluster that contained all of these experimentally identified centromeric H3 variants and some additional sequences that, based on best blast hits, were also likely to be orthologous to CenpA. The cluster did not contain the candidate orthologs in *Toxoplasma gondii* [81]. We do not know whether this is due to an error in the gene phylogeny, or to parallel invention of a centromeric H3 variants in this species, which would mean that it is not orthologous to CenpA. Nevertheless, we included these sequences in the orthologous group. The candidate centromeric H3 variants that are part of the CenpA cluster include sequences from all five eukaryotic supergroups: *Homo sapiens* [82], *Saccharomyces cerevisiae* [83], *Drosophila melanogaster* [84], *Caenorhabditis elegans* [85], *Schizosaccharomyces pombe* [86] (Opisthokonta), *Dictyostelium discoideum* [87] (Amoebozoa), *Arabidopsis thaliana* [88] (Archaeplastida), *Tetrahymena thermophila* [89], *Plasmodium falciparum* [90] (SAR), *Giardia intestinalis* [91] and *Trichomonas vaginalis* [92] (Excavata). The original gene tree in newick format is provided (Dataset EV3).

© 2017 The Authors

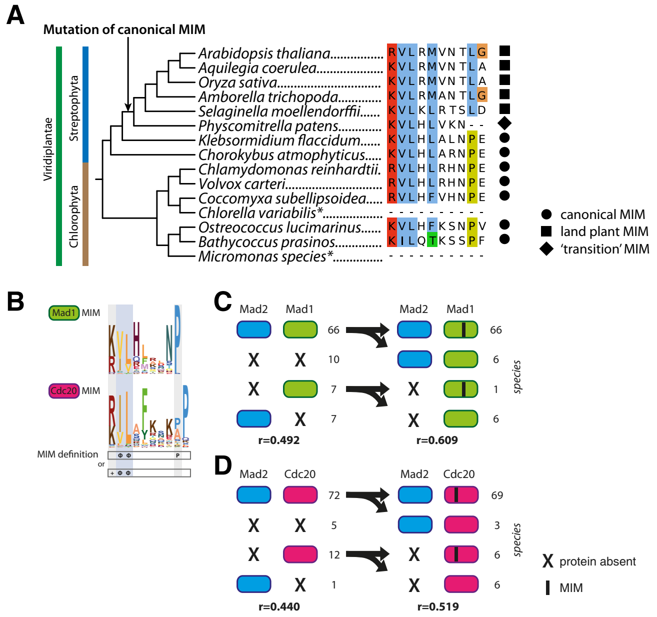

Figure EV5. Evolution of the Mad2-interacting motif (MIM) in green plants and co-occurrences of Mad2 with the MIM under a less strict motif definition.

A Viridiplantae (green plants) phylogeny [93] and the occurrences of the canonical MIM or the "land plant" MIM in Mad1 orthologs of the associated species. Asterisk (*) indicates species lacking an aligned MIM, possibly caused by incomplete gene prediction of Mad1 orthologs.

B The sequence logos of the MIMs of Mad1 (upper panel) and Cdc20 (lower panel) based on the alignments of the motifs present in the right-sided panels of (C and D). Below is indicated the required amino acid sequence of the MIM (+: positive residue, Φ: hydrophobic residue, P: proline). In contrast to Fig 6, the MIM is considered present if it agrees with the pattern [ILV](2)X(3,7)P or [RK][ILV](2), in order that the land plant motif suffices.

C, D Left side: Numbers of presences and absences of Mad2 in 90 eukaryotic species and its interaction partners Mad1 (C) and Cdc20 (D). Right side: Frequencies of Mad2 and MIM (according to definition in B) occurrences in species having Mad1 (C) or Cdc20 (D), respectively. Also the Pearson correlation coefficients (*r*) for the corresponding phylogenetic profiles are shown.

 © 2017 The Authors
